# Skimmin Improves Insulin Resistance *via* Regulating the Metabolism of Glucose: *In Vitro* and *In Vivo* Models

**DOI:** 10.3389/fphar.2020.00540

**Published:** 2020-04-29

**Authors:** Guoqiang Zhang, Xin Cai, Lingmin He, Dingmei Qin, Hongwen Li, Xiaoming Fan

**Affiliations:** ^1^School of Biotechnology and Food Engineering, Anyang Institute of Technology, Anyang, China; ^2^Department of Human Anatomy, College of Basic Medical Sciences, Guilin Medical University, Guilin, China; ^3^College of Pharmaceutical Science, Yunnan University of Chinese Medicine, Kunming, China

**Keywords:** skimmin, glucose metabolism, palmitic acid, high fat and high sugar, insulin resistance

## Abstract

Skimmin is the major pharmacologically active component present in *Hydrangea paniculata*, in the traditional Chinese medicine as an anti-inflammatory agent, and its anti-inflammation and anti-diabetic effect has had been studied in previous studies. The metabolism of glucose plays an important role in the pathophysiology of diabetes. Therefore, it was identified as an important target for improving diabetic. Herein, we found that skimmin relieved the palmitic acid and high-fat and high sugar-induced insulin resistance. Furthermore, skimmin enhanced the glucose uptake *via* inhibiting reactive oxygen species (ROS) and reducing the level of inflammatory correlation factor. Meanwhile, skimmin reduced the glucose output by promoting PI3K/Akt signaling pathway and down-regulating the expression of glycogen synthase kinase-3β (GSK3β) and glucose-6-phosphatase (G6Pase). In conclusion, skimmin can improve the insulin resistance by increasing glucose uptake and decreasing glucose output *in vitro* and *in vivo*.

## Introduction

Type 2 diabetes is a group of metabolic diseases which characterized by multiple etiologies caused chronic hyperglycemia. The main cause of type 2 diabetes is insulin resistance. Insulin resistance means the physiological doses of insulin cannot get their normal biological effects, and decrease the uptake and utilization of glucose (Sathya Bhama et al., 2012). The previous studies indicated that the number of people with diabetes in China have reached 92.4 million in 2010 (Yang et al., 2010). The people of diabetes will be reached 336 million in 2030 (Wild et al., 2004).

The establishment of insulin resistance model opens a window to study diabetes. Palmitic acid was usually used to induce the model of insulin resistance in HepG2 cells (Chen et al., 2019). Meanwhile, high fat and high sugar were utilized to induce the model of insulin resistance in SD rats (Fan et al., 2019a). With these models, it becomes more easier to research the effectiveness of drugs. Due to the low toxicity of natural drugs, more and more people are competing for prevention and treating type 2 diabetes.

Previous study showed that skimmin can improve membranous glomerulonephritis through suppressing inflammation and immune complex deposition (Zhang et al., 2013). Skimmin also can inhibit the streptozotocin-induced diabetic nephropathy in Wistar rats (Zhang et al., 2012). However, the molecular mechanism of skimmin for suppressing insulin resistance has not been reported before.

In our study, this is the first study to investigate the skimmin can increase glucose uptake, promote glycogen synthesis and improve insulin resistance *in vitro* and *in vivo*.

## Materials and Methods

### Cell and Animal Model Building

The HepG2 cells were purchased from ATCC, Virginia, USA. It was cultured in Dulbecco’s modified Eagle’s medium (DMEM) with 10% fetal bovine serum (FBS) at a 37°C incubator containing 5% CO_2_. HepG2 cells were exposed in different dose of palmitic acid (0.1, 0.2, 0.3, 0.4 mmol/L) for different time (24, 36, 48, 60 h). Then they were administrated with different concentration of skimmin (10, 20,40 μM) for 24 h. The glucose content of medium was tested by glucose oxidase kit. The optimal dosage and time of palmitate were determined according to the glucose concentration of medium.

Adult Sprague Dawley rats (6–8 W) (n = 50) were purchased in Laboratory Animal Center of Zhengzhou University. The experiment of animals was approved by the Institutional Animal Care and Use Committee of Anyang Institute of Technology (IACUC approval no. 2018-001). The animals were anesthetized with 10% chloral hydrate and sacrificed by cervical dislocation. All efforts were made to minimize suffering. At first, The animals were randomly divided into two group: normal diet group (n = 10) and HFHS diet group (n = 40). The normal group was basic diet. The HFHS diet group was feed with 60% basic diet, 20% lard, 15% refined sugar, 1.5% cholesterol, 0.1% sodium cholate, and 3.4% peanuts. After 12 weeks of dietary manipulation, the 40 rats were again randomly divided into 4 groups: HFHS group (n = 10), Low dose (10 mg/kg/d) skimmin group (n = 10), Middle dose (25 mg/kg/d) skimmin group (n = 10), High dose (50 mg/kg/d) skimmin group (n = 10). Each group was administrated for 4 weeks.

### Glucose, Insulin, and Hepatic Function Assay

The glucose concentration of medium and blood were detected by the kit of glucose oxidase-peroxidase (Baoping Bioengineering Institute, Zhengzhou). Glycogen levels in medium and liver tissue were measured *via* the kit of Glycogen Assay (Baoping Bioengineering Institute, Zhengzhou) according to the instructions of manufacturer. The levels of TNF-α, IL-6, insulin, and IL-1β were detected by ELISA kits (Baoping Bioengineering Institute, Zhengzhou) following the instructions of manufacturer. Alanine transaminase (ALT) and aspartate transaminase (AST) were determined by the automated biochemistry analyzer.

### MTS Assay

Skimmin was obtained from the Zhengzhou Baoping Biological Technology Co. LTD (Zhengzhou, China). The purity of skimmin is 98.5%. After the cells were treated with different concentration of skimmin, the cytotoxicity of skimmin was detected by MTS (3-(4,5-dimethylthiazol-2-yl)-5-(3-carboxymethoxyphenyl)-2H-tetrazdium) assay kit according to the instruction.

### DCFH-DA Staining Combined With Flow Cytometry Assay

The cells were seed (1*10^5^) in 6-well plates and cultured overnight, and then were fed with serum-free medium containing DCFH-DA (1:8000). Then, the cells were continuously cultured for 30 min in the incubator and washed with PBS for two times, the cells were collected and filtered with 200 using mesh screen. Then the intracellular ROS levels were determined by flow cytometry according to our previous research methods (Fan et al., 2019b).

### Western Blot

The protein of cells was extracted by RIPA lysate, and its concentration were determined by the BCA method. The samples were loaded to 10% SDS-PAGE gel and transferred onto polyvinylidene fluoride (PVDF) membrane. After the membrane was blocked by 5% skim milk, it was incubated with a specific primary antibody against p38 MAPK, JNKs, NF-κB, PI3K, Akt, G6Pase, phosphorylated p38 MAPK(Thr180/Thr182), phosphorylated JNKs (Thr182/Thr185), phosphorylated NF-κB (Ser 536), phosphorylated PI3K (Tyr458/Tyr199), phosphorylated Akt (Ser 473), and β-actin at 4°C overnights. All above antibody dilution concentration is 1:1000. Then, the membrane was incubated with the horseradish peroxidase (HRP)-conjugated secondary antibody (1:3000). The strip was visualized by enhanced chemiluminescence (ECL) kit and quantified using Image J 12.0 software.

### Immunohistochemistry Staining

The tissue sections (5 μm) was performed antigen retrieval by microwave after deparaffinization and rehydration for 10 min in sodium citrate buffer. Sections were cooled to room temperature, treated with 3% H_2_O_2_ for 10 min and blocked with 5% goat serum 40 min at room temperature. The sections were incubated at 4locked with 5% goat serum 40 min at room temperature. The sections weodium citrate buffer. Sections were cooled to room temperature, treated with 3% Hnt target for improving diabetic. Herei-rabbit, diluted 1:200) for 30 min. The sections were counterstained with hematoxylin after diaminobenzidine staining according to our previous research methods (Fan et al., 2019a).

### Statistical Analysis

Data were expressed as the mean ch methods 40 min at room temperature. The sections weodium citrate buffer. Sections were cooled to room temperature, treated with 3% Hnt target for improving diabetic. Heresis of variance. *P <* 0.05 was considered to indicate a statistically significant difference.

## Results

### The Model of Insulin Resistance Is Built *In Vitro* and *In Vivo*

**Figure 1:**
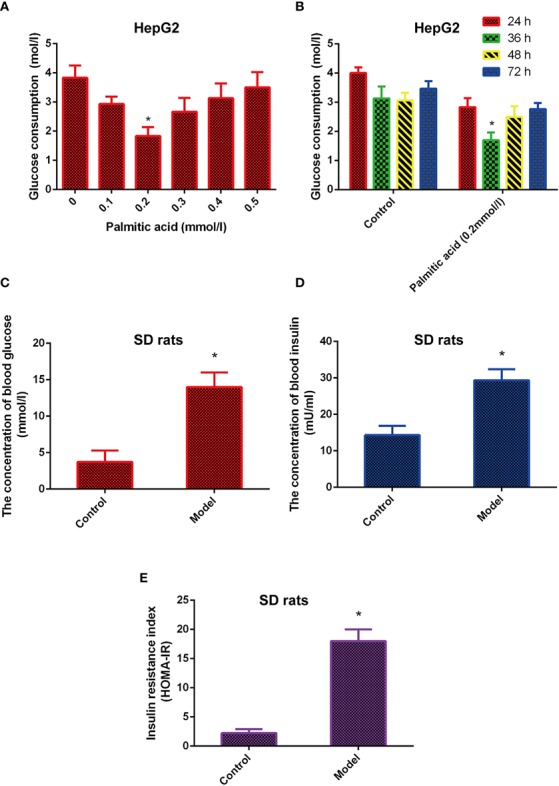
The model of insulin-resistance is built *in vitro* and *in vivo*. **(A)** Effects of different concentrations of palmitic acid on glucose consumption. **(B)** Effects of different time of palmitic acid on glucose consumption. **(C–E)**. High fat and high sugar induced the insulin resistance model *in vivo*. Insulin resistance index (HOMA-IR) were calculated by formula INS*GLU/22.5. ^*^Significant compared with control group alone, *P < 0.05*. LD, low dose; MD, middle dose; HD, high dose.

The cells model of insulin resistance was established using palmitic acid to induce HepG2 cells. The cells were treated in different dose of palmitic acid (0.1, 0.2, 0.3, 0.4, 0.5 mmol/l) for different time (24, 36, 48, 72 h). Next, the glucose content in medium was detected by glucose oxidase kit. The results indicated that the glucose consumption was highest in the 0.2 mmol/L of palmitic acid. In addition, when 0.2 mmol/L palmitic acid caused the highest blood glucose consumption at 36 h (**Figures 1A, B**). Therefore, the insulin resistance cell model was established under the following conditions: 0.2 mmol/L palmitic acid for 36 h.

Besides, the animal model of insulin resistance was established by high fat and high sugar. The results showed that the levels of glucose, insulin, and insulin resistance index (HOMA-IR) were increased in the model group compared with the control group (**Figures 1C–E**), which means the animal model of insulin resistance was built successfully.

### Skimmin Reduce Blood Glucose and Improve Insulin Resistance *In Vitro* and *In Vivo*

**Figure 2:**
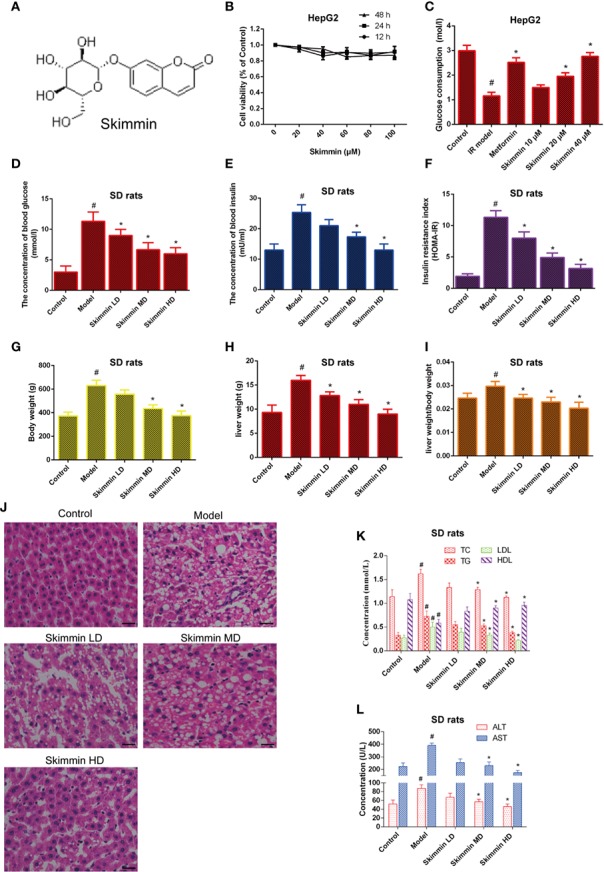
Skimmin reduce blood glucose and improve insulin resistance *in vitro* and *in vivo*. **(A)** The chemical structure of skimmin. **(B)** Cytotoxic effect of skimmin on HepG2 cells. **(C)** Skimmin promotes the uptake of glucose. **(D–F)**. Skimmin inhibits insulin resistance in a dose dependent manner. **(G–I)** Skimmin decrease the body weight and liver weight of rat induced by high fat and high sugar. **(J)** Skimmin attenuates the pathologic change of liver tissues. Bar = 20 μm. **(K)** Ginsenoside Rg1 reduces the concentration of serum lipid under the condition of insulin resistance. **(L)** Ginsenoside Rg1 improve the function of liver under the condition of insulin resistance. ^#^Significant compared with control group alone (*P* < 0.05). ^*^Significant compared with insulin resistance group alone (*P* < 0.05). TC, triglyceride; TG, total cholesterol; LDL, low-density lipoprotein; HDL, high-density lipoprotein; ALT, alanine transaminase; AST, aspartate transaminase.

The chemical structure of skimmin is shown in **Figure 2A**. MTS assay results showed that skimmin had no cytotoxicity to HepG2 cells (**Figure 2B**).

Then, we investigated whether skimmin had an effect on the glucose consumption of palmitic acid-induced HepG2 cells. The results showed that skimmin promoted the absorption of glucose in a dose dependent manner in palmitic acid-induced HepG2 cells. Metformin was used as the positive control group (**Figure 2C**).

Furthermore, the *in vivo* studies showed that skimmin decreased the level of serum glucose, insulin, and improved HOMA-IR (**Figures 2D**–**F**). Furthermore, we found that skimmin can decrease liver weight, body weight, and ratio of them induced by high fat and high sugar (**Figures 2G**–**I**). Besides, HE staining showed that skimmin inhibited the pathological changes of liver induced by high fat and high sugar (**Figure 2J**). Meanwhile, skimmin suppressed the secretion of lipid factors (**Figure 2K**), and improved the function of liver in a dose dependent manner (**Figure 2L**).

### Skimmin Increase the Uptake of Glucose by Reducing the Activation of Inflammatory Signaling and Inhibiting Oxidative Stress *In Vitro* and *In Vivo*

**Figure 3:**
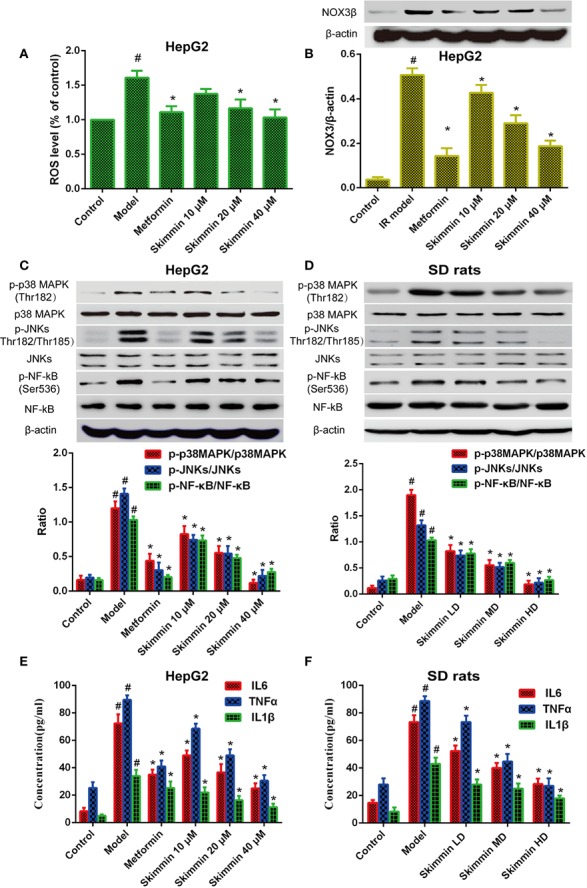
Skimmin increase the uptake of glucose by reducing the activation of inflammatory signaling and inhibiting oxidative stress *in vitro* and *in vivo*. **(A)** Insulin promote the production of ROS in insulin resistance HepG2 cells. **(B)** Skimmin inhibit the expression of NOX3 in a dose dependent manner. **(C**, **D)** Skimmin inhibit the phosphorylation expression of p38 MAPK, JNKs, and NF-κB in a dose-dependent manner *in vitro* and *in vivo*. **(E, F)** Skimmin inhibits the secretion of inflammatory cytokines under the condition of insulin resistance *in vitro* and *in vivo*. The values shown are mean SEM of data from three independent experiments. ^#^Significant compared with control group alone, *P* < 0.05. ^*^Significant compared with model group alone, *P* < 0.05. ROS, reactive oxygen species; NOX3, NADPH oxidase.

Nowadays, we have known that skimmin can promote the uptake of glucose to improve insulin resistance *in vitro* and *in vivo*. However, the molecular mechanism by which skimmin reduce blood glucose is still unclear. Oxidative stress is the pathological basis of insulin resistance (Cremonini and Oteiza, 2018; Dos Santos et al., 2018). The activity of the NADPH oxidase (NOX) is critical to the production of ROS in the organism. Previous studies showed that the ROS production increased by up-regulating the expression of NADPH oxidase 3 (NOX3), activating p38MAPK and JNK signaling pathway, and inducing insulin resistance in palmitate-induced HepG2 cells (Gao et al., 2010; Malik et al., 2019). Our studies found that skimmin can inhibit palmitic acid-induced the production of ROS, and 40 μmol/L skimmin was more obvious using flow cytometry assay (*P <* 0.05), which was better than metformin, a drug used to treat diabetes (**Figure 3A**). In addition, skimmin also inhibited the increased of NOX3 protein compared with the insulin resistance group induced by palmitic acid. The effect of 40 µM skimmin was better than metformin (**Figure 3B**) (*P <* 0.05). What is more, we found that skimmin reduced the phosphorylation expression of p38MAPK and JNKs compared with insulin resistance group in a dose dependent manner *in vitro* and *in vivo* (**Figures 3C, D**). AP-1 transcription factors, including c-Fos, c-Jun, and ATF, which also are the down-stream of p38 and JNKs, have a well-known role in promoting IL-6 and TNF-α transcription (Oh et al., 2013; MacNeil et al., 2014). In order to further detect the anti-inflammatory mechanism of skimmin, we tested the expression of NF-κB and the secretion of inflammatory factors. Our studies indicated that skimmin inhibited the phosphorylation level of NF-κB and the secretion of IL-6, IL-1β, and TNF-α in a dose dependent manner *in vitro* and *in vivo* (**Figure 3E, F**). The above results showed that skimmin can promote the uptake of glucose and reduce inflammation caused by insulin resistance through reducing the production of ROS and suppressing the expression of NOX3, p-p38MAPK, p-JNKs, and NF-κB.

### Skimmin Improve the Synthesis of Liver Glycogen by Increasing the Phosphorylation of Akt and Inhibiting the Expression of GSK3β *In Vitro* and *In Vivo*

**Figure 4:**
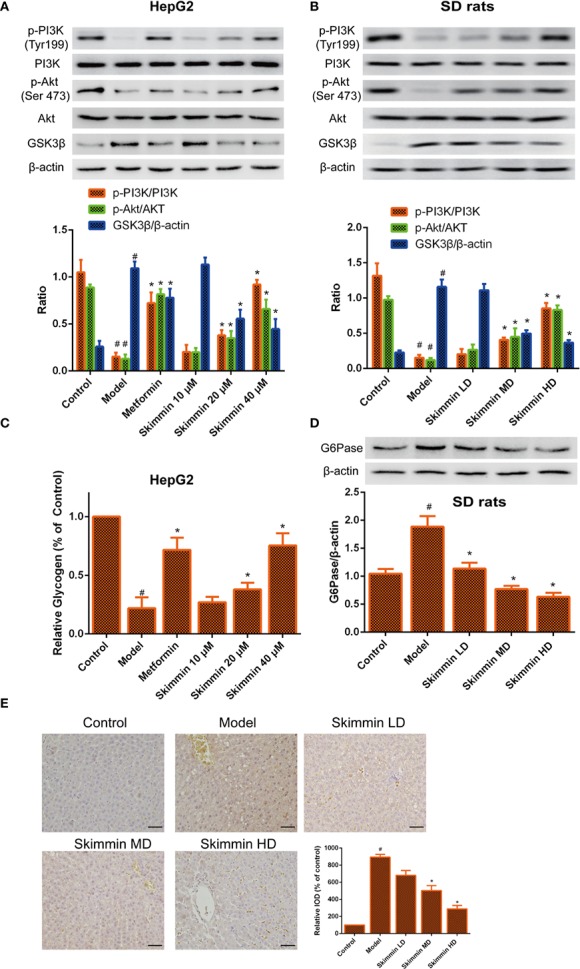
Skimmin increase the phosphorylation of Akt, inhibit the expression of GSK3β, and increase the synthesis of liver glycogen *in vitro* and *in vivo*. **(A**, **B)** Skimmin upregulate the protein expression of phosphor-PI3K, phosphor-Akt and downregulate the protein expression of GSK3β in a dose dependent manner *in vitro* and *in vivo*. **(C)**. Skimmin increases the synthesis of glycogen in a dose dependent manner *in vitro*. **(D**, **E)** Skimmin inhibits the expression of G6Pase in a dose dependent manner *in vivo*. Bar = 20 μm. ^#^Significant compared with control group alone (*P* < 0.05). ^*^Significant compared with insulin resistance group alone (*P* < 0.05). GSK3β, glycogen synthase kinase-3β; G6Pase, glucose-6-phosphatase.

GSK3β is a critical enzyme which can reduce the synthesis of liver glycogen, increase the concentration of blood sugar in the body (Zhang et al., 2018). Some studies indicated that Grifola Frondosa stimulates glycogen synthesis by regulating PI3K/Akt/GSK3 signaling pathway, improving insulin resistance (Ma et al., 2014). To further explore the relationship between skimmin and glycogen synthesis. The PI3K/Akt/GSK3 signaling pathway was determined by Western blot after skimmin treatment *in vitro* and *in vivo*. The results demonstrated that skimmin can obviously upregulate the level of p-PI3K, p-Akt and downregulate the level of GSK3β compared with insulin resistance model group in a dose dependent manner *in vitro* and *in vivo* (**Figures 4A, B**).

Previous studies showed that GSK3β inhibitor can suppress the synthesis of glycogen (Maqbool and Hoda, 2017). Furthermore, the volume of glycogen was detected by glycogen assay kit. We found that glycogen was decreased in the model group, while skimmin can increase the volume of glycogen in a dose dependent manner *in vitro* (**Figure 4C**). G6pase increases the blood glucose concentration in the body by promoting the decomposition of glycogen (Chou et al., 2015). Immunohistochemical and Western blot results demonstrated that skimmin inhibit the expression of G6pase in a dose dependent manner *in vivo* (**Figures 4D, E**).

The above results showed that skimmin can decrease glucose output by up-regulating the phosphorylation level of PI3K and Akt, suppressing the expression of GSK3, promoting the synthesis of glycogen. Meanwhile, skimmin also can decrease the expression of G6pase, inhibit the breakdown of glycogen.

## Discussion

The decomposition of glycogen can increase blood glucose, which is then decomposed and used by cells. The excess blood glucose is converted into glycogen, maintaining the balance of blood glucose in the body under the regulation of insulin and glucagon. Under the condition of insulin, insulin of normal concentration cannot play its due role, thus causes the increase of blood sugar (Yang et al., 2011; Tangvarasittichai, 2015). We explored the molecular mechanism of insulin resistance from two aspects: the utilization of blood glucose and the decomposition of glycogen.

Oxidative stress leads to the production of a large number of ROS, which further leads to the apoptosis of islet cells, and then cause insulin resistance (Hu et al., 2016; Kuzmenko et al., 2016; Alcalá, et al., 2017). Previous studies showed that TNF-α induced the expression of NOX3, promotes ROS production, activates the JNKs signaling pathway, and produces insulin resistance in the HepG2 cells (Walton, 2017). What is more, high sugar and ROS also can activate p38MAPK and induce insulin resistance in vascular smooth muscle (Liu et al., 2018). Therefore, it is necessary to find the agent to decrease ROS, suppress the secretion of inflammatory factors. enhance islet cell secreting function, increase the utilization of blood glucose and reduce blood glucose.

In addition, the glycogen synthesis also plays a critical role in the glucose output of the liver. Previous studies indicated that the enhancement of glycogenesis and decrease in glycogen synthesis are also responsible for insulin resistance (Ren et al., 2018). The PI3K/Akt/GSK3 signaling pathway is involved in the metabolism of glycogen. The activation of PI3K is required for insulin-stimulated glucose uptake (Yang et al., 2015). Phosphorylated Akt can inhibit the activity of GSK3, and GSK3 can suppress synthesis of glycogen by inhibiting glycogen synthase (Yang et al., 2017). Furthermore, G6pase is a critical enzyme in gluconeogenesis and glycogenolysis and plays an important role in the homeostasis of glucose (Chou et al., 2015). Inhibiting the expression of G6pase can also reduce blood glucose. So, it is also important to find drugs to inhibit the production of glucose through upregulating the expression of p-AKt, downregulating GSK3 expression, and inhibiting G6pase expression.

**Figure 5:**
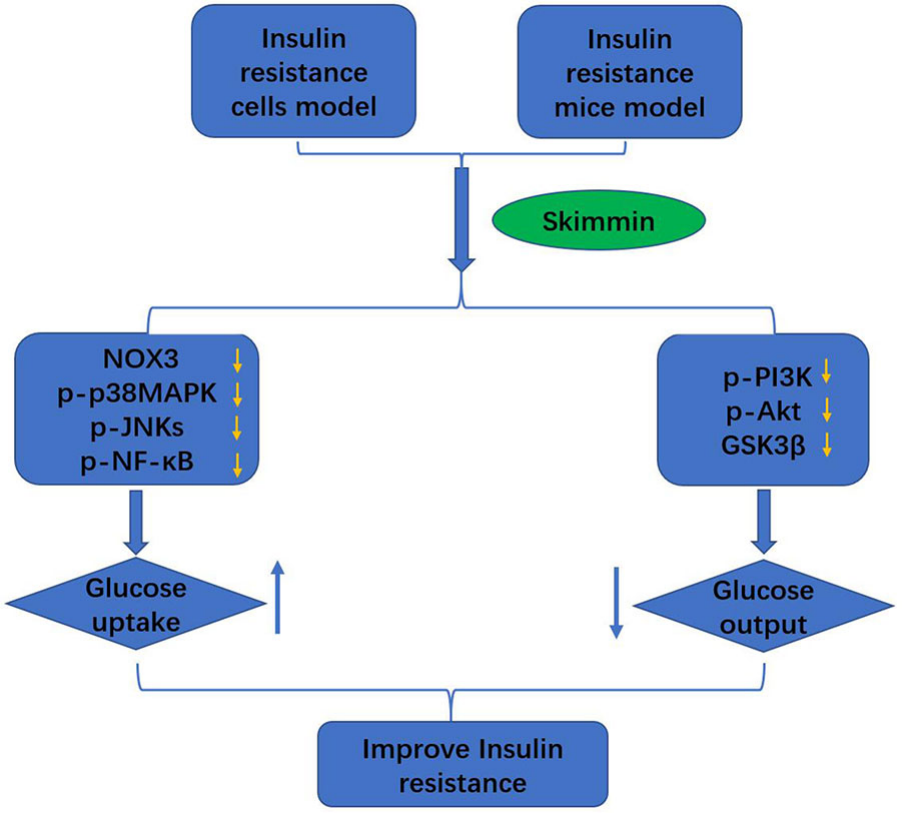
Skimmin improve the insulin resistance by promoting the glucose intake, and inhibiting the glucose output.

Skimmin is the main active substance in Hydrangea paniculata, and has the characteristics of anti-inflammation, anti-plasmodial, and anti-cancer (Moon et al., 2011). We build the palmitic acid-induced HepG2 insulin resistance cell model and high fat and high sugar-induced insulin resistance SD rat model. Then skimmin was used to treat the HepG2 cells and SD rats, metformin as a control agent. The results showed that skimmin increased glucose intake and suppressed the inflammatory response through decreasing the production of ROS, suppressing the protein expression of NOX3, p-p38MAPK, and p-JNKs. Meanwhile, skimmin decreased glucose output by increasing the phosphorylated PI3K and Akt, suppressing the expression of GSK3β, reducing the glycogen synthase, increase liver glycogen synthesis, and inhibiting G6pase expression, reducing decomposition of glycogen (**Figure 5**). The above will provide better drug options for the treatment of type 2 diabetes. To validate the utility of skimmin for type 2 diabetes patient improvement, more detailed investigation regarding pharmacokinetics of skimmin following its overdose should be addressed. In addition, the effectiveness of skimmin on the relevance markers of insulin resistance need to be further explored.

Taken together, skimmin improve eventually the insulin resistance by promoting the glucose intake, and inhibiting the glucose output.

## Data Availability Statement

All datasets generated for this study are included in the article/supplementary material.

## Ethics Statement

The animal study was reviewed and approved by Anyang Institute of Technology.

## Author Contributions

XF and HL designed the research, GZ and XC performed the research and wrote the paper. LH and DQ analyzed the data.

## Conflict of Interest

The authors declare that the research was conducted in the absence of any commercial or financial relationships that could be construed as a potential conflict of interest.

## Abbreviations

ALT, alanine aminotransaminase; AST, aspartate transaminase; MAPK, mitogen-activated protein kinase; NF-κB, nuclear factor kappa-B; ERKs, extracellular regulated protein kinases; JNKs, c-Jun N-terminal kinases; PI3K, Phosphoinositide-3 kinase; NOX3, NADPH oxidase 3; GSK3H, glycogen synthase kinase-3o; G6pase, glucose-6-phosphatase; MTS, 3-(4,5-dimethylthiazol-2-yl)-5-(3-carboxymethoxyphenyl)-2H-tetrazdium; DCFH-DA, 2′,7′-Dichlorofluorescin diacetate.
